# Research Participants’ Perspectives on Using an Electronic Portal for Engagement and Data Collection: Focus Group Results From a Large Epidemiologic Cohort

**DOI:** 10.2196/18556

**Published:** 2020-10-01

**Authors:** Erika Rees-Punia, Alpa V Patel, Asher Beckwitt, Corinne R Leach, Susan M Gapstur, Tenbroeck G Smith

**Affiliations:** 1 American Cancer Society Atlanta, GA United States; 2 Asher Consulting Atlanta, GA United States

**Keywords:** focus groups, health information technology, epidemiologic studies

## Abstract

**Background:**

Epidemiologic cohort studies have begun to leverage electronic research participant portals to facilitate data collection, integrate wearable technologies, lower costs, and engage participants. However, little is known about the acceptability of portal use by research participants.

**Objective:**

The aim of this study is to conduct focus groups among a sample of Cancer Prevention Study-3 (CPS-3) participants to better understand their preferences and concerns about research portals.

**Methods:**

CPS-3 participants were stratified based on sex, race and ethnicity, age, and cancer status, and randomly invited to participate. Focus groups used an exploratory case design with semistructured guides to prompt discussion. Using a constant comparison technique, transcripts were assigned codes to identify themes.

**Results:**

Participants (31/59, 52% women; 52/59, 88% White/non-Latinx) were favorably disposed toward using a research participant portal to take surveys, communicate with the study staff, and upload data. Most participants indicated that a portal would be beneficial and convenient but expressed concerns over data safety. Participants stressed the importance of an easy-to-use and trustworthy portal that is compatible with mobile devices.

**Conclusions:**

In addition to being beneficial to researchers, portals may also benefit participants as long as the portals are secure and simple. Participants believe that portals can provide convenient ways to report data and remain connected to the study.

## Introduction

### Background

A common, contemporary application of health information technology is the electronic patient portal [1]. Electronic patient portals are secure web-based systems that provide patients 24-hour access to their personal health information, enable two-way communication with health care providers, and potentially decrease costs through patient self-service [2,3]. Currently, less than half of US adults use patient portals annually, although use is more common among younger (younger than 65 years), more educated people with broadband internet access [4,5]. Patient portals also have the potential to facilitate the integration of new technologies for remote monitoring, such as home blood pressure monitors, ecological momentary assessment apps, and other wearable devices (eg, activity trackers and heart rate monitors) at a lower cost [6]. Collectively, patient portals can simplify the collection of patient-generated health data (PGHD), including vital signs (eg, temperature, blood pressure, and blood glucose), lifestyle data (eg, caloric intake, diet, exercise, and medication adherence), and patient-reported outcomes data (eg, mood, sleep quality, and pain) [7].

The use of electronic health portals has spread beyond the clinical setting. The adoption of commercial products such as Apple HealthKit [8], 23andme [9], and PatientsLikeMe [10] illustrates that an increasingly large number of people are willing to exchange medical and health behavior information with professionals other than their health care providers. Electronic portals have also been leveraged to survey and engage participants in research studies. Similar to the various benefits of patient portals, research participant portals could facilitate the collection of PGHD and data from wearable technologies, provide a way to administer targeted surveys to different subsets of the study population, collect electronic health data, and save costs and time associated with printing and mailing logistics [11]. Portals may be useful for frequently engaging participants in long-term studies, potentially reducing attrition. In addition to the aforementioned researcher benefits, participants may find portals convenient for storage and anytime access to study materials such as copies of consent forms, surveys, and study newsletters or updates.

Despite the potential benefits, very little is known about the feasibility and acceptability of electronic portal use by research participants in longitudinal cohort studies. Participant portals have been adopted by a few prospective cohorts thus far, including the University of California-San Francisco’s PRIDE study [12] and the National Institutes of Health All of Us study [13]; however, none of them have published details regarding their portal development and testing. A better understanding of research participants’ preferences and concerns about research portals could inform portal design and maximize the benefits to and engagement of participants and researchers.

### Objectives

In this study, focus groups were implemented among a sample of Cancer Prevention Study-3 (CPS-3) participants to facilitate the open sharing of insights largely unavailable through pre-existing surveys, especially (1) research participants’ perceptions of the facilitators and barriers to utilizing an electronic research portal; (2) participants’ views about using an interactive research portal to submit health records, complete lifestyle and medical surveys, and communicate with study staff; and (3) participants’ recommendations for electronic research portals.

## Methods

### Sample Recruitment

CPS-3 is a contemporary prospective epidemiologic cohort study initiated by the American Cancer Society (ACS) [14]. In total, 303,682 participants aged 30-65 years across the United States, including Puerto Rico, were enrolled in CPS-3 between 2006 and 2013. Participants are sent surveys approximately every 3 years to update exposure and medical information and will be followed to identify incident cancers and cause-specific mortality through linkages with state cancer registries and the National Death Index, respectively. The Emory University Institutional Review Board approved all aspects of CPS-3.

In 2017, plans to develop a CPS-3 participant portal began as a way to increase participant engagement and facilitate survey administration. This effort launched with a series of virtual and on-site focus groups conducted to gain insight into participant perspectives and willingness to share data through a participant portal. Virtual focus groups, offered via conference calls, enabled the inclusion of a broader geographic distribution of CPS-3 participants. To be eligible for virtual focus groups, participants must have been involved with CPS-3 since enrollment (2006-2013) and must have (1) a valid email address and (2) completed the 2018 triennial CPS-3 survey online. On-site focus groups took place in Atlanta, Georgia, and Albany, New York, and allowed participants who had not previously engaged with CPS-3 online to be included. To be eligible for on-site focus groups, participants must have (1) completed the 2018 survey (online or paper) and (2) resided in one of the predefined zip codes near the on-site locations.

Eligible CPS-3 participants were stratified based on sex, race and ethnicity, age group, and cancer status and invited to participate in the portal feasibility focus groups. We used this purposive sampling strategy to ensure adequate representations in focus groups by race and ethnicity, sex, age, and cancer history. Overall, 608 participants were invited, 78 registered to participate, and 59 participated in the focus groups.

### Study Design

The focus groups used an exploratory case design, which is used when there are no clear or predictable outcomes [15,16]. To guide the focus group discussion, semistructured guides were developed before conducting focus groups and were used to prompt group discussions regarding perceived portal benefits, barriers, and preferences (Multimedia Appendix 1). All focus group participants were assigned a pseudonym for anonymity and were asked to introduce themselves using their pseudonym before sharing opinions to ease transcription.

### Qualitative Analysis

All focus groups were audio recorded and transcribed verbatim, excluding phrases like *um* and *uh*. A ground-up or grounded theory approach was used to analyze the data [17]. Open coding was initially performed to code each line of the text. During this process, in vivo codes were generated based on the participants’ words. Following the open coding process, the data were coded using axial coding. The open-coded data were iteratively and systematically compared and then linked together using one or more codes. Finally, selective coding was used to identify the core categories that emerged from the data. Selective coding was completed by re-reading the data and coding data that related to these categories.

Coding consensus was used to validate the data. One researcher coded all data, and two additional researchers reviewed all codes and their assignment to the text; any differences in coding assignments were adjudicated until consensus was reached. NVivo 12 (QSR International) was used to store, code, query, and organize all data [18].

## Results

### Sample Characteristics

A total of 11 focus groups were conducted in November 2018, with 7 virtual focus groups (2-8 participants each) and 4 on-site (6-8 participants each). Of the 11 focus groups, 5 consisted entirely of participants sharing an underrepresented demographic characteristic (1 focus group of all African Americans, 1 virtual and 1 on-site focus group of all older adults [60 years and older], and 1 virtual and 1 on-site focus group of all cancer survivors). The focus group participant (n=59) demographics are presented in Table 1. About half of the participants were women (31/59, 52%) and most were non-Latinx White (52/59, 88%). The age range of participants was 41 to 72 years (mean 59, SD 6 years), with about half over the age of 60 years (32/59, 54%).

**Table 1:** Individual-level demographics (n=608 invited, n=59 participants).

Characteristic		Participants invited (n=608)		Participants registered (n=78)		Focus group participants (n=59)	
Women, n (%)		314 (51.6)		41 (52)		31 (52)	
**Race and ethnicity, n (%)**							
	Non-Latinx White		458 (75.3)		65 (83)		52 (88)
	Black		50 (8)		5 (64)		3 (5)
	Latinx		50 (8)		6 (7)		2 (3)
	Other		50 (8)		2 (2)		2 (3)
**Age group, n (%)**							
	59 years and under		507 (83.4)		62 (80)		27 (46)
	60 years and older		101 (16.6)		16 (21)		32 (54)
Cancer survivor, n (%)		100 (16.4)		11 (14)		8 (15)	
**Focus group location, n (%)**							
	Virtual		406 (67)		46 (59)		31 (52)
	On-site		202 (33)		32 (41)		28 (48)

As an introduction to the topic of electronic portals, participants were asked about their current portal use. Most, although not all, of the participants were familiar with the concept of online portals, and many already use them with their health care providers and/or banks. On the other hand, there were 3 on-site and 2 online focus group participants who stated they “have no experience with online portals,” “don’t know” if they have access to one or have ever used one, or “have not set up” a portal. Most participants described the benefits and barriers to general portal participation, including improved communication with health care providers, appreciation for 24-hour access to health care records, and concerns about private health information remaining secure. These benefits and barriers were also consistent with the existing literature on patient portals [19-23].

### Benefits of Research Portals

The majority of participants indicated that a research participant portal would be beneficial. Participants cited convenience as the main benefit of portal use. For example, one participant described the convenience of the all-hours access:

...being able to access 24/7 is very helpful. It's the convenience, and then also you get two-way feedback. It's more interactive.

Other benefits (Textbox 1, theme 1) include appreciation for having all their study material at one place:

...I would say convenience, being able to access [the portal] on my phone...all my data is in one place. I don't have to keep a lot of paper that I'm worried about getting in the wrong hands.

**Additional quotes by theme.**

**Theme 1: Perceived portal benefits**

Subtheme: convenience

1. “Same thing with the [CPS-3 study] newsletters, you can go back and access, but a lot of us read them and then we possibly throw it out or it ends up in a pile somewhere. We don't remember where it is. I think that's going to be a great advantage of having the portal.”
2. “I would like having it all in that one portal. It's there, it's handy. You can go back. If I travel, I have the app on my phone. I can pull it up, it's there”

**Theme 2: Portal use concerns**

Subtheme: worried about data privacy

1. “I volunteered to do this study for discovery of treatment or diagnoses to better peoples’ health. I think that should be limited to that only. ... it's not happening in health data yet, but the selling of stuff to other people. That kind of thing should not happen.”
2. “...what nags me about the online portals is, how much information am I giving out? Who is actually seeing this? And how do I know it's secure, whether it's coming or going?”

Subtheme: not concerned about data privacy

1. “I'm okay with my information being used for educational purposes and by colleges and by universities and students and professors for purposes of figuring out what's going on to heal cancer patients and stuff like that.”

**Theme 3: Information accessed or shared through the portal**

Subtheme: comfortable sharing information

1. “I don't mind sharing all that information with the American Cancer Society in relation to this study, and I wouldn't mind my doctor sharing it directly with them. I have no qualms with any of that.”
2. “...the whole point like somebody said, we chose to be participants in this study to gain information, so I would think you would want, and need said information. I'm fine. If I wasn't fine, I wouldn't participate in the study in the first place, so I'm fine sharing that information with them. Again, presuming it's all confidential...”

Subtheme: not comfortable sharing certain information

1. “I'm okay with sharing most information, especially regarding research. The one piece of information I probably would not be as comfortable with is anything that relates to sex.”
2. “I think it's hard for people to share any kinds of mental health or certain diagnoses, and I think cancer, or any kind of chronic illness might be that. Because if you need to find health insurance, sometimes I think there is a feeling that it's going to be...people are going to know it, and you're not going to be able to get insurance, or life insurance. If somehow, they can access this information.”

**Theme 4: Communication with the American Cancer Society staff**

Subtheme: comfortable communicating

1. “The ease of both ways communication with using a portal what strikes me if it's on my screen, I get a message, or I'm asked a question, I'm likely to respond. If I get something in the mail or that's going to get lost in the shuffle.”
2. “I'm going to look at what the letters are after the name. Does it say RN? Is the person who's answering my question properly trained to have answered my question? That would be my concern, not whether it's John Smith, RN, but whether it's RN or not. In terms of knowing who all the people are who are involved, when would we ever know that? That doesn't seem relevant to me, whereas the qualifications of the person to respond matter a lot.”

**Theme 5: Cancer Prevention Study-3 (CPS-3) portal desires**

Subtheme: CPS-3 study information

1. “...any successes where the researchers have used our data to help point them at some conclusions that are going to have an impact, certainly we've all gotten in to this so that we would have an impact and if we can be rewarded with milestones, it would be wonderful.”
2. “I'd like to see how many people participate and how many of these poor people have gotten cancer in the meantime and how long it's been going on and all that.”

Subtheme: security

1. “That two-factor authentication gives me a much more better comfort level.”

Subtheme: portal-based surveys

.

1. “...if I know ahead of time that the survey's gonna take longer then I'm probably gonna be okay with it... but if I start a survey and I don't know how long it's gonna take and it's going on forever, I'll quit it.”
2. “I would want to know... can I save it and stop and come back to it? And/or is there any information that I need other than just my memory to take this survey, or to think about before I actually start taking it?”

Subtheme: technical support

1. “I personally find online customer service chats to be way more effective and easier than phone calls. You have a transcript, too, afterwards when you're done.”
2. “I don't really perceive needing any assistance, just the basic. Being able to call an 800 number or chat with somebody online if there was a difficulty.”
3. “I must have 35 passwords. Between remembering capital, star, or whatever it is, space and all that stuff and if you're not exact then you don't get in. If you're only doing it once every three months, it's difficult. It just needs to be easily you can retrieve a password.”

### Research Portal Use Concerns

Participants’ concerns were focused largely on data privacy, including data storage and use (Textbox 1, theme 2). For example, many participants felt uninformed about how their data would be used and by whom:

I would be concerned as to how long that data would be stored. I mean if the study's only going to be 20 years, what happens to the data at the end of that 20 years?

Several participants also specifically mentioned concerns about their data being accessed by employers or insurance companies, impacting their careers and/or insurability:

I think that in light of the pre-existing condition kind of controversy out there, I would have the same concerns that my insurance company would get that information and use that somehow against me.

A few participants shared that they generally do not trust the internet, with one explaining:

I don't know that much about the internet and the ethernet or whatever they call it, but who knows who could get a hold of... my medical records going across the airwaves, I don't know.

Conversely, some participants did not feel worried about data privacy. One participant reported the following:

I don't know that I have concerns. I was assuming the information would be used for research in order to help prevent cancer...I'm trusting.

### Information Accessed or Shared Through the Research Portal

Many participants expressed willingness to share certain types of information through portals, such as CPS-3 survey responses, medical records, and/or data from electronic devices (eg, wearable physical activity monitors and blood pressure monitors; Textbox 1, theme 3). When prompted, a few participants said they were willing to "share whatever [researchers] want". Another participant stated:

One of the reasons we signed up for this study was we wanted to give information so that it could be used for future generations... I have no problem sharing everything.

On the other hand, several participants expressed reluctance to share information they perceived as more sensitive, such as details regarding their mental health or sexual habits (Textbox 1, theme 3, subtheme: not comfortable sharing certain information, quote 1). One participant shared:

I think anything with mental health, there’s still a stigma there. So, if I have [mental health] issues... I would not feel comfortable sharing.

Participants were also hesitant about providing too many personal identifiers on a portal:

The things I don't feel comfortable sharing, I guess on health-related sites, would be my social security number, my Medicare number, things that other people can use to really get access to information about me and use in other harmful ways.

Several participants drew a clear distinction between patient and research participant portals. They expressed a willingness to share details on patient portals but set limits on the amount of information they would share on research participant portals. One participant described the conditions required for sharing medical information with researchers:

I'm a little concerned about keeping a clear distinction between researchers and medical providers...I'm willing to provide some of my medical records to researchers as long as I'm clear as to what they're doing with that and how they're asking that I transfer any kind of medical information aside from just surveys.

In contrast, other participants clearly understood the distinction between patient and research participant portals and felt equally willing to share their information through both:

I think the portal for our personal physician is a different situation. There it helps the patient/physician interaction and helps save time for both parties. As far as a portal for this study or studies like this, I think it's very important to assist research. I'm a big fan of sharing information in an anonymous fashion to help the research parties involved.

### Communications With Study Staff

When asked, most participants expressed a willingness to receive and respond to messages through the participant portal (Textbox 1, theme 4). Further, participants felt that two-way communication would improve study engagement, as exemplified in this participant’s thoughts:

I would like to feel more connected to the study... I don't even know how often we get the opportunity to participate in answering a survey. And sometimes I don't feel connected to it. I feel like, “Have I missed one because I missed that email?”...more communication with the ACS in regards to the study might help me feel more connected to it.

These perspectives were echoed by other participants:

I think the communication piece is really important and even as we take surveys, now I sometimes find certain questions not as clear as I would like them to be, it would give us that avenue to offer good feedback to the team working on it in the first place.

However, as another participant noted, some understanding of the study staff credentials would ease communication:

I want to know a little bit about who's responding, so if the responder has some certification or some specialty in whatever the information they're providing, that would be really helpful to know. I don't want to take information on face value unless I know that it's coming from someone who has a background, at least, and experience.

### Features of a Research Portal

Participants expressed a desire for a “well-designed [portal] that is user-friendly” and “doesn’t have a lot of bells and whistles.” Participants made several specific recommendations for features and content they would like to see on a research participant portal. For example, several participants hoped to find information regarding participant demographics (Textbox 1, theme 5), study design, and research findings on the portal:

I'd like to see some updates, some CPS-3, some just in general, regarding the work they're doing. Then, also, maybe a section for comments or questions.

Participants also spent time discussing their wish for a safe and secure portal, with several participants advocating specifically for two-step verification:

I like a two-step validation or verification process...while it is a little inconvenient because it requires extra steps is, if I'm going to log onto a portal, the portal will send a message to my cell phone with a code and then to complete the login, I have to use the code. I like that kind of two-step authorization.

Most participants expressed openness to completing regular surveys through the portal. During these discussions, the topic of preferred survey length frequently arose, with several participants stating that they were willing to commit “as long as it takes” or however much “time [the surveys] require.” However, other participants had specific suggestions for maximum survey length and administration frequency:

I think 15 to 20 minutes for a survey is an appropriate amount of time for me and how often, two or three times a year I think would be okay.

Other participants suggested providing clear indicators of survey length (Textbox 1) by adding a completion bar or page countdown to help them manage their time while taking more lengthy surveys:

I'm more likely to finish the survey if during it, it tells you how far you are so that you know if you're getting close to the end or not.

Finally, many participants discussed the importance of technical support (Textbox 1, theme 5). Participants expressed concerns over having too many passwords and reiterated the importance of some sort of technical support to quickly retrieve a new password:

I would like just a quick password reset email if necessary. Something quick as opposed to 20 minutes later and perhaps an online video if things get very technical.

## Discussion

### Principal Findings

In this qualitative study, we investigated epidemiologic cohort research participants’ perspectives and expectations of an electronic portal for study engagement and data collection. Through 11 focus groups, we found that most participants were favorably disposed toward the concept of using a portal to participate in the cohort study as long as the portal is secure and easy to use. Many themes that arose in this study were consistent across groups. Importantly, we were able to recruit enough participants to gain an understanding of how research participants in longitudinal cohort studies may view the implementation of an electronic portal. Given that the final focus group did not produce any significant new information, we can assume that we achieved data saturation. This suggests that participants in CPS-3 and other longitudinal cohort studies might be willing to use a research portal that has the potential to benefit both researchers and participants.

Some of the themes that arose in this study regarding research participant portals were similar to those documented in the literature regarding health care patient portals. For example, the most commonly discussed perceived benefit in this study was convenience, especially regarding access to research records anywhere, at any time, with a variety of electronic devices, which also applies to health care portals [19,20]. Similarly, the most commonly cited concern was data privacy and protection, including hacking, data sharing, and password management [20,24]. Although participants were informed of data privacy and protection at the time of CPS-3 enrollment, continuing to educate and inform participants is clearly important, and a participant portal would make it easier to do so. Given the similarities in the themes presented here and in patient portal research, it is possible that some findings regarding the design and implementation of patient portals may be applied to research portals. This is potentially important as there is considerably more research on the use of portals for health care purposes than on the use of portals for research purposes.

Several novel concepts relating specifically to research participant portals have emerged. Generally, participants were willing to upload medical records, connect wearable devices, and share other PGHD through an electronic portal outside of a health care setting. Although most participants were open to completing surveys on the portal, many wished to be informed of the survey duration before and/or during administration. Similarly, participants expressed their preference to save responses and complete surveys at a time that is convenient for them. Participants’ willingness to provide survey data appears to be based primarily on the altruistic belief that the data could benefit others through research findings. Although altruistic motivation for research participation may not be a novel concept [25], it is useful to understand these motivations when considering how to communicate the importance of logging in and participating on the portal. Finally, most participants were willing to communicate with the study staff through the portal, although several participants mentioned the importance of understanding the staff member’s credentials.

On the basis of the participant feedback provided through the focus groups, we recommend that research participant portals be easy to use and function on all platforms (eg, mobile phones, tablets, and all commonly used web browsers). Given participant concerns regarding data privacy and protection, it is essential to design a portal with robust security (eg, dual-factor authentication) and easy access to data privacy policies from the signed informed consent form. Research participant portals should also provide timely technical support. Surveys administered through a portal should include progress bars and options to save and return to the survey later. The findings also suggest that a portal would be more appealing to participants if it connects and engages them through information on new study findings and discoveries.

### Strengths and Limitations

This study has some limitations. First, the level of transferability (ie, the degree to which these findings can be applied to other groups or initiatives) of these results is unclear, particularly as the participants in this study have already demonstrated a long-term commitment to a longitudinal cohort study. Additionally, although several underrepresented groups were present in our focus groups, we did not have large enough subgroup sample sizes to consider differences by group. Similarly, only 2 participants were present for one of the online focus groups, although more had initially registered; thus, group discussion may have been affected for that particular group. Although it is beyond the scope of this study, more consideration around the real-world integration of electronic research portals is needed, especially as participants presumably begin to gain access to more electronic patient portals through their health care providers. Future research should identify ways to simplify the implementation and management of research portals for both participants and study staff. Despite these potential limitations, this study is, to our knowledge, the first to systematically investigate the views of research participants regarding online research participant portal use.

### Conclusions

Participant portals may be beneficial to researchers for facilitating new data collection, integrating wearable technologies, administering targeted surveys on special topics to subset populations, saving costs and time compared with mailed surveys, and engaging participants between survey cycles. For participants, portals have the potential to reduce burden by providing convenient ways to report data, review study materials, and remain connected to the study. The rich data from these focus groups provide a rationale for building a research participant portal and information to inform the design of the portal. To achieve these potential benefits, portals should be easy to use, secure, trustworthy, and compatible with a variety of devices.

## Appendix

Multimedia Appendix 1 Semistructured focus group guide.

## Abbreviations

ACS: American Cancer Society
CPS-3: Cancer Prevention Study-3
PGHD: patient-generated health data
