# Supplementary material for: Research Participants’ Perspectives on Using an Electronic Portal for Engagement and Data Collection: Focus Group Results From a Large Epidemiologic Cohort
Source: J Med Internet Res. 2020 Oct 1;22(10):e18556. doi: 10.2196/18556 (PMC7563628; doi:10.2196/18556)
Supplement: Multimedia Appendix 1 [file jmir_v22i10e18556_app1.docx]

CPS-3 Focus Group Guide (General Questions)

# Introduction

My name is XXX and I work for Asher Consulting, LLC. We are conducting focus groups on behalf of the American Cancer Society (ACS) to investigate CPS-3 participants willingness and opinion of utilizing an online portal. By “portal” we mean a website where you would be able to provide CPS-3 related information and communications in one place, similar to the website you may use to do your banking. The information we collect from you during these focus groups will be used to inform if and what type of information you would like to see ACS staff provide and what information you might be willing to share through an online portal specifically designed for CPS-3 participants. We know you are very busy, and we appreciate the time you are spending with us today to share your ideas and thoughts.

This focus group should take about 60-90 minutes and is voluntary. We will be audio recording and transcribing the session. We will protect your confidentiality by using the pseudonyms we previously assigned to you during our session today and in attributing specific comments in the transcription of this session. When all the focus groups are completed, we will create a summary report of the findings for ACS staff to help them better understand participants willingness and perceptions of utilizing an online portal, and ultimately in guiding how to design that portal. This is your opportunity to lend your expertise to that process.

Here are some “ground rules” for participation: a) please use your pseudonym prior to speaking. For example, please state “This is Amy” and then complete your response, b) please allow one person to speak at a time, c) please be respectful of each other and do share participants responses with people outside of this group d) please make sure you are in a quiet environment with no background noise (online only), and e) please mute yourself when you are not speaking (online only). As the moderator, I will start the discussion and keep us on time. But I am not an expert in the topic. You are the experts. I am simply leading the group**.**

Do I have your permission to record the focus group? Do you have any questions before we get started?

# Interview Guide

*Icebreaker:* **Before we get started, let’s introduce ourselves. Please tell us the name that was assigned to you during our discussion and tell us your favorite hobby. Prompt (if necessary): This could be exercising, cooking, or watching sports.**

# The first few questions will help us understand your willingness to use an online portal. After these questions, we will talk about your thoughts about the specific information captured through the portal.

## What is your opinion of using the internet to access private information (e.g. credit cards, banking, or health information)? What do you think?

## Tell me about any experiences you may have in using online portals to access health information.

## Probe: What did you use it for?

## Probe: What information did you access using the online portal?

## Probe: What information did you share using the online portal?

d. Tell me about any types of information you were not comfortable sharing?

## What do you perceive are the benefits to using participant portals?

## Probe: What are the benefits of using an online portal to communicate with ACS staff?

## Probe: What are the benefits of using online portals to access health information (e.g. diagnosis, diet, and exercise)?

## Describe any barriers or concerns you may have in using online portals. Please tell me any concerns you may have about: (*Note: Facilitator, please ask these one-by-one.)*

## Probe: Hackers/password security

## Probe: Identity theft

## Probe: Personal health data security

## Probe: Storage and use of data

## Probe: Not knowing the staff responding to your messages

## Probe: Seeing medical information in writing

- 1. Probe: Not understanding the medical information provided online (e.g. a laboratory or pathology report)
  2. Probe: The quality of communication with ACS staff
  3. Probe: Technical difficulty in uploading electronic information from a device like an activity monitoring device (e.g. Fitbit)
  4. Probe: Anything else we did not already cover?

# Next, I am going to ask for your opinions about using online portals to *access* your health information.

## How do you feel about using an online portal to access and share information with CPS-3 staff about: (*Note: Facilitator, please ask these one-by-one.)*

- 1. Probe: Health information regarding your diagnosis and treatment
  2. Probe: Information about your lifestyle (e.g. sleep, diet, exercise)

## For this question, we are going to ask you about your level of comfort in using technology to locate medical information from your Primary Care or other doctors. Please describe your comfort level in using technology to:

- 1. Find and download your medical records from your patient portal? (If they experience problems ask, what are the challenges in locating this information?)

## Now I am going to ask you about your opinion of using a portal to *share* your information. Describe your level of comfort in using the portal to: (*Note: Facilitator, please ask these one-by-one.)*

- 1. Probe: Communicate with ACS staff/providers
  2. Probe: Take online surveys
  3. Probe: Upload your personal medical records
  4. Probe: Upload your personal lifestyle information (e.g. sleep patterns, foods that you eat, physical activity (e.g. Fitbit data)

# We are now going to ask you a series of questions about your recommendations for portal use.

## What would you want to see in a CPS-3 participant portal?

- 1. Probe: The ability to share your personal medical and lifestyle information through medical records or devices or syncing health-related apps (e.g. sleep patterns and physical activity through your Fitbit)
  2. Probe: Communicate with ACS staff/providers
  3. Probe: Take surveys

## Tell me about any recommendations you may have to help motivate you and other participants to sign up to use the CPS-3 online portal?

- 1. Probe: How do you feel about receive regular emails with reminders about signing up to use the portal?

## What do you recommend to help encourage you and other participants to regularly use the CPS-3 online portal? (*Note: Facilitator, ask these one-by-one)*

- Probe: How do you feel about receiving regular emails reminding you to use the portal?
- Probe: Do you have any other suggestions to encourage you utilize the portal?
- Probe: Describe how frequently are you willing to be contacted to perform any tasks (e.g. monthly, quarterly, semiannually, annually)?

## Describe the level of technical support you may need to facilitate using an online CPS-3 participant portal. Describe any help you may need regarding:

- 1. Probe: Assistance with initial login
  2. Probe: Toll-free assistance hotline/email technical support
  3. Probe: Anything else?

## Is there anything else you would like to tell us about your perceptions or recommendations for portal use, or anything important that we missed?

# Conclusion

I want to thank you for participating in the focus group today and for sharing your experiences and perceptions. The information you have provided will be very helpful in informing the next steps regarding portal development.
